# MEASURING SOCIAL ISOLATION AMONG OLDER ADULTS WITH DEMENTIA: TRENDS, CHALLENGES, AND NEXT STEPS

**DOI:** 10.1093/geroni/igae098.0805

**Published:** 2024-12-31

**Authors:** Mary Louise Pomeroy, Mfon Umoh, Yiqing Qian, Katherine Ornstein, Thomas Cudjoe

**Affiliations:** Johns Hopkins University, Baltimore, Maryland, United States; Johns Hopkins University School of Medicine, Baltimore, Maryland, United States; Johns Hopkins University, Baltimore, Maryland, United States; Johns Hopkins University, Baltimore, Maryland, United States; Johns Hopkins University School of Medicine, Baltimore, Maryland, United States

## Abstract

A growing body of literature demonstrates a strong association between social isolation and the risk of incident dementia. However, research examining objective social isolation among persons living with dementia (PLWD) has been limited. The behavioral and psychological symptoms of dementia may reduce social connection. Research that uses population-based, national surveys to examine this issue among PLWD must consider missing data and proxy interview responses. We report new evidence on the prevalence of social isolation among a national sample of community-dwelling PLWD and offer strategies to attenuate missing proxy data. Using 2015 data from the National Health and Aging Trends Study and a multi-domain measure of social isolation that considers living arrangement, core discussion network size, religious attendance, and social participation, we identified that 41.3% (n=313/757) of interviews with PLWD were completed using a proxy respondent. In proxy interviews, one of the four social isolation items is not asked. We classified social isolation status when a sum score based on the three remaining items equaled 0 (socially isolated; n=19/313) or 2 or 3 (not socially isolated, n=99/313). This strategy resolved 37.7% (n=118/313) of the missing proxy data and reduced missing social isolation data across PLWD to 26.0% (197/757). About 32.1% (n=180/560) of PLWD were socially isolated, irrespective of proxy status. Social isolation was more prevalent among PLWD who completed the interview with no proxy (36.4%; n=161/442) compared to those with a proxy (16.1%; n=19/118) (X2(1)=17.6; p<.001). Future work should consider imputation strategies when assessing social isolation in PLWD using proxy interviews.

